# A phase 1 dose escalation study of the oncolytic adenovirus enadenotucirev, administered intravenously to patients with epithelial solid tumors (EVOLVE)

**DOI:** 10.1186/s40425-019-0510-7

**Published:** 2019-01-28

**Authors:** Jean-Pascal Machiels, Ramon Salazar, Sylvie Rottey, Ignacio Duran, Luc Dirix, Karen Geboes, Christine Wilkinson-Blanc, Gillian Pover, Simon Alvis, Brian Champion, Kerry Fisher, Hilary McElwaine-Johnn, John Beadle, Emiliano Calvo

**Affiliations:** 1 Department of Medical Oncology, Institut Roi Albert II, Cliniques universitaires Saint-Luc and Institut de Recherche Clinique et Expérimentale, Université catholique de Louvain, Brussels, Belgium; 2 Medical Oncology Department, Catalan Institute of Oncology, IDIBELL, University of Barcelona, Barcelona, Spain; 3 Drug Research Unit Ghent, Ghent University Hospital, Ghent, Belgium; 4 Instituto de Biomedicina de Sevilla, IBiS/Hospital Universitario Virgen del Rocío/CSIC/Universidad de Sevilla, Seville, Spain; 5 Saint-Augustinus Hospital, Antwerp, Belgium; 6 Department of Gastroenterology and Digestive Oncology, Ghent University Hospital, Ghent, Belgium; 7 PsiOxus Therapeutics Limited, 4–10 The Quadrant, Barton Lane, Abingdon, UK; 8 Department of Oncology, University of Oxford, Oxford, UK; 9 START Madrid, Centro Integral Oncológico Clara Campal, Hospital Madrid Norte Sanchinarro, Madrid, Spain

**Keywords:** Clinical trials, Pharmacokinetics and pharmacodynamics, Enadenotucirev, Oncolytic adenovirus, Epithelial solid tumor, Intravenous

## Abstract

**Background:**

Enadenotucirev is a chimeric adenovirus with demonstrated preclinical tumor-selective cytotoxicity and a short half-life. Further clinical mechanism of action data showed that enadenotucirev can gain access to and replicate within different types of epithelial tumors. This phase 1 dose escalation study assessed intravenous (IV) dose escalation with enadenotucirev to establish the maximum tolerated dose (MTD) and subsequently identify a suitable schedule for repeated cycles.

**Methods:**

Sixty-one patients with advanced epithelial tumors unresponsive to conventional therapy were enrolled and received enadenotucirev monotherapy as part of this study. During the phase 1a dose escalation (*n* = 22) and expansion (*n* = 9), delivery of enadenotucirev between 1 × 10^10^ and 1 × 10^13^ viral particles (vp) on days 1, 3, and 5 (single cycle) was used to determine an appropriate MTD. Subsequent treatment cohorts (phase 1a, *n* = 6 and phase 1b, *n* = 24) examined the feasibility of repeated dosing cycles in either 3-weekly or weekly dosing regimens.

**Results:**

Enadenotucirev displayed a predictable and manageable safety profile at doses up to the MTD of 3 × 10^12^ vp, irrespective of infusion time or dosing schedule. The most commonly reported treatment-emergent adverse events (TEAEs) of grade 3 or higher were hypoxia, lymphopenia, and neutropenia. The frequency of all TEAEs (notably pyrexia and chills) was highest within 24 h of the first enadenotucirev infusion and decreased upon subsequent dosing. Additionally, delivery of three doses of enadenotucirev over 5 days optimized pharmacokinetic and chemokine profiles in the circulation over time.

**Conclusions:**

This study provides key clinical data in patients with solid epithelial tumors following treatment with IV enadenotucirev monotherapy and supports further investigation of enadenotucirev in combination with other therapeutic agents at doses up to the MTD of 3 × 10^12^ vp.

**Trial registration:**

(ClinicalTrials.gov Identifier: NCT02028442). Trial registration date: 07 January 2014 – Retrospectively registered.

**Electronic supplementary material:**

The online version of this article (10.1186/s40425-019-0510-7) contains supplementary material, which is available to authorized users.

## Background

The use of oncolytic viruses is rapidly emerging as a novel therapeutic approach against cancer [1–4]. Such viruses selectively infect tumor cells, directly killing infected cells as well as initiating systemic antitumor immune responses [5, 6]. Several oncolytic viruses are being tested in clinical trials [7], either as monotherapies or as part of multimodal therapeutics (e.g. in combination with immune checkpoint inhibitors and chemotherapy) to synergize their activity and improve outcomes for patients [2, 5].

Systemic delivery is a major goal in the field of oncolytic viruses [4]. Intravenous (IV) delivery of oncolytic viruses offers advantages over intratumoral (IT) injection, including the opportunity to target inaccessible tumors and to treat both the primary tumor and any overt or undiagnosed metastatic deposits simultaneously [8]. To date, talimogene laherparepvec, an attenuated herpes simplex virus type-1, is the only oncolytic virus therapy to be licensed by the European Medicines Agency and the US Food and Drug Administration for the local treatment of unresectable melanoma [9–11]. However, talimogene laherparepvec is administered by IT injection, restricting its use to accessible tumors and requiring specialist skills, particularly when treating non-superficial tumors.

Enadenotucirev is a group B Ad11p/Ad3 chimeric oncolytic adenovirus generated by directed evolution that has potent and tumor-selective cytotoxicity, inducing a non-apoptotic immunogenic death process [12, 13] and has been shown to have antitumor efficacy in orthotopic human tumor xenograft models [12, 14]. Furthermore, the original derivation of the virus from a colorectal cell line, the high level of activity in a range of colorectal cell lines and in vivo [12] as well as findings from a parallel clinical study [15], highlighted gastrointestinal and genitourinary malignancies as specific indications of interest. Enadenotucirev was prioritized for clinical development and delivery by IV infusion on the basis of preclinical evidence of its stability in human whole blood [16, 17] and the low prevalence of neutralizing antibodies against group B adenoviruses, including Ad11p (the outer coat of enadenotucirev is exclusively the Ad11p serotype), in the general population [18, 19].

Enadenotucirev is being tested in several trials in an international clinical program. One of these studies (the Mechanism of Action [MOA] study) demonstrated that enadenotucirev can be successfully administered by IV infusion or IT to a range of epithelial tumor types with a predictable and manageable safety profile [15]. This study also demonstrated that enadenotucirev gained access to and replicated within tumors, and was associated with high levels of CD8+ T-cells within the tumor nests [15].

We now report the primary findings of the phase 1 EValuating OncoLytic Virus Efficacy (EVOLVE) study. EVOLVE was designed to assess the safety and tolerability of enadenotucirev dose escalation administered by IV infusion in patients with colorectal cancer (CRC) and other solid epithelial tumors to determine the maximum tolerated dose (MTD), as well as selecting a suitable schedule for repeat cycle IV infusion in patients with metastatic CRC (mCRC) or urothelial cell carcinoma (UCC).

## Methods

### Study design and dosing schedules

**Fig. 1:**
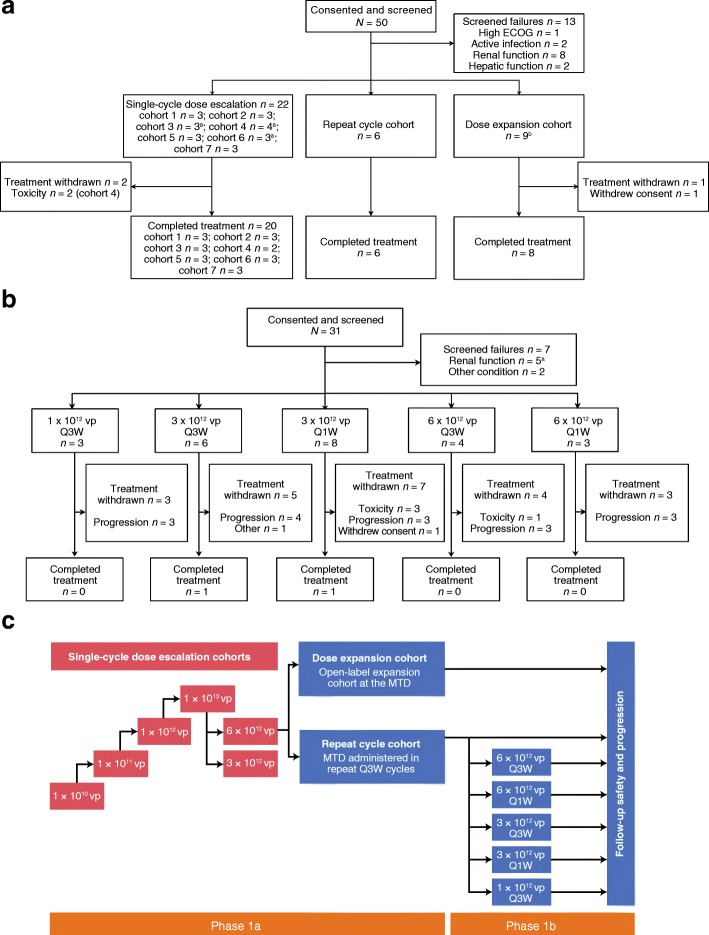
Patient disposition and study design. The phase 1 single-cycle dose escalation and dose expansion stages of EVOLVE were designed to determine the MTD of enadenotucirev, given in one cycle (one administration on days 1, 3, and 5 and an end-of-study visit 56 days after the last administration of enadenotucirev). **a** The phase 1a repeat cycle stage comprised up to four 21-day cycles and an end-of-study visit 28 days after the last administration of enadenotucirev. **b** The phase 1b component was initiated to select a suitable schedule and dose for repeat cycles of enadenotucirev in patients with mCRC or UCC. Phase 1b comprised up to six 21-day cycles, ending with an end-of-study visit 28 days after the last administration of enadenotucirev. Please refer to the study design and dosing and patient enrollment sections of the methods and patient demographics section of the results for full details of the enrollment into this phase. **c** Overall study design of the EVOLVE study. *CRC* colorectal cancer, *ECOG* Eastern Cooperative Oncology Group, *EVOLVE* EValuating OncoLytic Virus Efficacy, *IV* intravenous, *mCRC* metastatic colorectal cancer, *MTD* maximum tolerated dose, *Q1W* weekly schedule, *Q3W* 3-weekly schedule, *UCC* urothelial cell carcinoma, *vp* viral particle(s). ^a^One participant later received one additional cycle of treatment. ^b^Three participants received one or more additional cycles of treatment. ^c^One participant also had inadequate bone marrow function

The EVOLVE study was a phase 1 (NCT02028442), multicenter, non-randomized, open-label study to investigate the administration of enadenotucirev monotherapy in patients with advanced or metastatic epithelial solid tumors. The EVOLVE study design is shown in Fig. 1.

The study consisted of single-cycle dose escalation and dose expansion cohorts and a repeat cycle cohort (phase 1a), as well as additional optimal schedule-finding repeat cycle cohorts (phase 1b). Key study decisions were taken by centralized study decision-making committees. Safety and tolerability data (as well as supporting laboratory data) were reviewed at each point of study treatment adjustment by a Clinical Events Committee (CEC). Safety data were reviewed by a Data and Safety Monitoring Committee (DSMC) at the end of phase 1a. Patients were followed up in phase 1a for 9 months and in phase 1b for overall survival.

All patients were screened in the 21 days before the start of study treatment. The starting dose was 1 × 10^10^ viral particles (vp) infused over 5 min, determined to allow a 40-fold safety margin below the no observed adverse effect level from the relevant Good Laboratory Practice toxicology study. Three patients were enrolled sequentially with at least a 14-day window and followed up during the dose-limiting toxicity (DLT) assessment period (28 days after the first enadenotucirev administration). If no DLTs were observed, the next cohort was enrolled at a 10-fold higher dose. Expansion of a cohort, from three to six patients, was required if one of the three patients experienced a DLT at a given dose. If two or more participants had a DLT at a given dose, then no further patients received this dose and the previous lower dose level was defined as the MTD. Adverse events (AEs) that met any of the following criteria were considered DLTs if they were treatment-related and confirmed to meet the DLT criteria by the CEC.

- Grade 3 or higher non-hematological toxicity lasting more than 3 days despite optimal supportive care, except for:
  - self-limiting or medically controllable toxicities (e.g. nausea, vomiting, diarrhea, fatigue, headache, chills, electrolyte disturbances, hypersensitivity reactions) and alopecia.
- Not all enadenotucirev doses were administered over the 5 days owing to AEs considered treatment-related (cycle 1 only).
- Febrile neutropenia (the occurrence of an absolute neutrophil count < 0.5 × 10^9^/L concurrently with a temperature elevation of > 38.5 °C lasting more than 5 days).
- Grade 4 neutropenia or thrombocytopenia lasting more than 14 days.

In the dose expansion stage, the number of patients treated at the MTD, determined by the CEC, was expanded to 12 patients. The repeat cycle stage cohort was introduced to evaluate the safety and tolerability of repeated 3-weekly (Q3W) treatment cycles of enadenotucirev monotherapy. This cohort was initiated once six patients had been followed up for at least 28 days after the day 5 administration of enadenotucirev in the dose expansion stage. Phase 1b was initiated once six patients had been treated in the phase 1a repeat cycle stage to further assess repeated Q3W and weekly (Q1W) dosing schedules. A summary of the dosing regimens used during EVOLVE is shown in Additional file 1: Table S1.

### Patient enrollment

Patients were eligible if they were aged 18 years or over with an Eastern Cooperative Oncology Group performance status of 0 or 1 and predicted life expectancy of 3 months or more with adequate renal, hepatic, and bone marrow function and coagulation test results. Eligibility also required patients to have a gap of at least 3 weeks since their last dose of IV systemic chemotherapy and 2 weeks since their last oral dose of capecitabine, with recovery to grade 1 from the effects (excluding alopecia) of any previous therapy. Patients had to meet one of the following specific inclusion criteria for the different parts of the study.

- Dose escalation stage (in phase 1a): solid tumor of epithelial origin not responding to standard therapy or for which no standard treatment exists.
- Dose expansion and repeat cycles stages (phase 1a and phase 1b): mCRC not responding to standard therapy and no more than three previous lines of systemic therapy for advanced disease, or no more than four previous lines of systemic therapy for advanced disease if one of the four lines was an anti-epidermal growth factor receptor therapy given as a single agent or combined with a previously administered chemotherapy regimen. In phase 1b, patients with advanced or metastatic UCC who had received no more than one chemotherapy-containing regimen and one other systemic biologic agent were also included.

Key exclusion criteria included any condition or illness that would compromise safety or interfere with the evaluation of the safety of the study treatment, significant immunodeficiency, splenectomy, previous allogeneic or autologous bone marrow or organ transplantation, active infections or viral disease, use of anti-viral agents in the last 14 days, major surgery in the last 4 weeks or radiotherapy in the last 3 weeks, another primary malignancy in the last 3 years (except for non-melanoma skin cancer or cervical cancer in situ), and a central nervous system metastasis that was symptomatic and/or required treatment.

### Clinical assessments

Demographic and baseline data were collected at screening. The incidence, nature, and severity of AEs were characterized using the National Care Institute Common Terminology Criteria for Adverse Events (CTCAE) Version 4.03. Additionally, changes over time and maximum changes in laboratory data were analyzed.

Although antitumor activity was not the primary outcome of the study, the best overall response to enadenotucirev was monitored using the Response Evaluation Criteria in Solid Tumors (RECIST) Version 1.1, as assessed both by the investigator and by an independent reviewer. Tumor imaging was performed every 8 weeks (phase 1a) or every 9 weeks (phase 1b) until disease progression.

### Kinetics of enadenotucirev

The concentration of enadenotucirev in the blood was measured using quantitative polymerase chain reaction (qPCR) to detect genomic viral DNA as described previously [15]. Whole blood samples were collected during phase 1a on days 1, 3, and 5 pre-infusion, at the end of infusion (EOI), and at regular intervals for up to 6 h after EOI. Additional samples were obtained 24 h after EOI (on days 2, 4, and 6) and on days 8, 15, 22, and 61. During phase 1b, samples were taken pre-infusion, at EOI, and at regular intervals for up to 4 h after EOI on days 1 and 5 during cycles 1 and 2 of the Q3W schedule and days 1, 5, 8, and 15 for the Q1W schedule. Samples were also collected on all other dosing days pre-infusion and at EOI.

### Analysis of enadenotucirev shedding

The concentration of shed enadenotucirev was quantified via qPCR as described previously [15]. During phase 1a dose escalation, scheduled clinical samples were collected daily from baseline to day 6 and then on days 8, 15, 22, and 61. During dose expansion, samples were collected at baseline and on days 3, 5, 8, 22, and 61. During phase 1b, samples were collected on days 1, 5, 8, and 15 of cycles 1 and 2 and subsequently on days 1, 8, and 15 of cycles 3–6, and at the end of treatment during both dosing schedules. Shedding assays were performed on buccal and rectal samples in phase 1a, and urine samples in both phase 1a and phase 1b.

### Anti-enadenotucirev antibody response

Serum anti-enadenotucirev antibody response was assessed using a Meso Scale Discovery (Meso Scale Diagnostics, Rockville, MD, USA) enzyme-linked immunosorbent assay, as described previously [15]. During phase 1a, dose escalation and dose expansion stages, samples were collected pre-infusion on day 1 and subsequently on days 8, 15, 22, 61, and 120. During the Q3W schedule of phase 1b, samples were collected pre-infusion on days 1 and 5 of cycles 1 and 3–6, days 1, 3, and 5 of cycle 2, as well as at the end of treatment and follow-up visits. Samples were collected pre-infusion during cycle 1 (days 1, 5, 8, and 15) and subsequently on day 1 of cycles 2–6, as well as at the end of treatment and follow-up visit as part of the Q1W schedule.

### Viral infectivity assay

Blood samples were collected at EOI on each dosing day. 100 μL of blood samples were added to monolayers of HT-29 cells along with negative and positive controls. The extent of infection was scored depending on viral staining and monolayer destruction. If no staining of cells or plaque formation was observed, the sample was scored as negative.

### Cytokine responses

Cytokine levels were assessed using a FlowCytomix assay as part of phase 1a as previously described [15], and a Luminex bead-based multiplex assay (R&D Systems, Abingdon, UK) in phase 1b. During phase 1a dose escalation, serum samples were collected immediately before infusion, 6 and 12 h post-infusion on days 1, 3, and 5, 24 h post-infusion, and on day 8 (72 h post-day 5 infusion). For dose expansion, samples were collected 6 and 12 h post-infusion on days 1, 3, and 5, as well as on day 8. In phase 1b, samples were collected pre-infusion and 6 h post-infusion, with an option to collect additional samples 12 h post-infusion on all dosing days.

### Enadenotucirev replication in tumor tissue

Hexon staining was assessed using immunohistochemistry with a pan hexon monoclonal antibody (ab8251, Abcam, Cambridge, UK) as previously described [15].

### Statistical analysis

Viral kinetic, pharmacodynamic, and safety data were analyzed using descriptive statistics, frequency counts, and percentages, and were also shown graphically, if appropriate. Individual means were tested using Student’s *t*-tests and multiple means were compared using Tukey-Kramer honest significant difference analyses. A post hoc analysis categorized treatment-emergent adverse events (TEAEs) and laboratory test results by dose level (< 1 × 10^12^ vp, 1–3 × 10^12^ vp, and >  3 × 10^12^ vp). For this *post hoc *analysis, coding of all AEs was updated to the latest version of the Medical Dictionary for Regulatory Activities, Version 19.1.

## Results

### Patient demographics, disposition

Patients were aged between 36 and 79 years, were primarily men (75.4%), and all were Caucasian. During phase 1a, 50 patients were screened for study eligibility between September 24, 2012 and March 28, 2014 (Fig. 1a), of whom 37 were enrolled in the study and received the study treatment. As part of the phase 1 dose escalation, 22 of 24 patients (91.7%) received one cycle of treatment (one administration on days 1, 3, and 5), with the study treatment being withdrawn in two patients because of DLTs at the highest dose tested (1 × 10^13^ vp). One patient withdrew consent during the dose expansion stage of phase 1a, with eight of nine patients (88.9%) completing the treatment period. All patients (6/6) completed the repeat dosing cycle treatment period in phase 1a.

Phase 1b screened 31 patients between November 3, 2014 and March 29, 2016 (Fig. 1b); 24 were enrolled and received enadenotucirev. Twenty patients (83.3%) received all doses in cycle 1, 15 patients (62.5%) received all doses in cycle 2, and 13 patients (54.2%) received all doses in cycle 3. Three patients continued treatment beyond cycle 3, with one patient receiving all doses until cycle 5 day 8 on the Q1W schedule and two patients (8.3%) completing all six cycles of treatment, one on each of the dosing schedules. During treatment, 22 patients discontinued treatment because of disease progression (16 patients), toxicity (four patients), or withdrawal of consent from study treatment or investigator decision due to an unrelated AE concurrent with disease progression (one patient each).

### MTD and safety

Having increased the dose in 10-fold increments starting at 1 × 10^10^ vp/5 min, no DLTs were elicited at doses up to 1 × 10^12^ vp. Two DLTs were experienced in response to the 1 × 10^13^ vp/5 min dose (cohort 4). One patient developed hypoxia and dyspnea and a second acute lung injury (CTCAE grade 3); both DLTs were considered treatment-related and resulted in treatment discontinuation after the first dose. The dose investigated was subsequently de-escalated to 3 × 10^12^ vp (cohorts 5 and 6) and later re-escalated to 6 × 10^12^ vp (cohort 7). The infusion durations of cohorts 6 and 7 were increased to provide an infusion rate comparable to cohort 3, and the MTD was determined to be 6 × 10^12^ vp/40 min.

However, after initiation of phase 1b and following review of the additional safety data generated in this part of the study, the CEC and DSMC recommended reducing the dose for both dosing schedules (Q3W and Q1W) to 3 × 10^12^ vp/15 min owing to the overall frequency and severity of TEAEs (including severe hypoxia and raised hepatic transaminases) across the two initial phase 1b cohorts at the 6 × 10^12^ vp/40 min dose. At the end of the study, the investigators considered the MTD to be 3 × 10^12^ vp, irrespective of infusion time or dosing schedule.

Most patients experienced TEAEs during the first week of treatment, usually within 24 h of dosing. The overall incidence of TEAEs was generally higher during cycle 1 than during cycle 2. Preferred terms describing primary influenza-like symptoms (pyrexia, chills, and influenza-like illness) were among the most commonly reported, by 45 (73.8%), 41 (67.2%), and 13 (21.3%) patients, respectively. All chills and influenza-like illness events were grade 1 or 2, and only two patients (receiving 6 × 10^12^ vp) experienced grade 3 pyrexia, with none being considered a serious AE or leading to dose discontinuation (Table 1). A greater number of these TEAEs relating to influenza-like symptoms were reported within 24 h of the first cycle 1 dose than after the second or third doses, and the overall frequency was higher following the first dose in cycle 1 than following the initial doses of cycle 2.

**Table 1:** TEAE^a^ summary following enadenotucirev infusion

Category	Preferred term	Number (%) of patients reporting AEs				Number of TEAEs grade ≥ 3 (all dosing groups)
		Dose assigned (vp)				
		< 1 × 10^12^ vp (*n* = 6)	1–3 × 10^12^ vp (*n* = 26)	> 3 × 10^12^ vp (*n* = 29)	All dosing groups (*N* = 61)	
Any TEAE		6 (100) **[4]**	26 (100) **[25]**	29 (100) **[29]**	61 (100) **[58]**	161 **[90]**
Any SAE		1 (16.7)	7 (26.9) **[4]**	12 (41.4) **[6]**	20 (32.8) **[10]**	
TEAEs of interest^b^						
Influenza-like symptoms	Chills, *n* (%)	0	18 (69.2) **[18]**	23 (79.3) **[23]**	41 (67.2) **[41]**	0
	Influenza-like illness, *n* (%)	0	7 (26.9) **[7]**	6 (20.7) **[6]**	13 (21.3) **[13]**	0
	Pyrexia, *n* (%)	2 (33.3) **[1]**	20 (76.9) **[19]**	23 (79.3) **[23]**	45 (73.8) **[43]**	2 **[2]**
Acute respiratory symptoms	Acute lung injury, *n* (%)	0	0	1 (3.4) **[1]**	1 (1.6) **[1]**	1 **[1]**
	Dyspnea, *n* (%)	1 (16.7)	6 (23.1) **[2]**	7 (24.1) **[3]**	14 (23.0) **[5]**	3 **[2]**
	Hypoxia, *n* (%)	0	1 (3.8) **[1]**	5 (17.2) **[5]**	6 (9.8) **[6]**	9 **[9]**
	Interstitial lung disease, *n* (%)	0	0	1 (3.4) **[1]**	1 (1.6) **[1]**	1 **[1]**
Renal events	Acute kidney injury, *n* (%)	0	1 (3.8)	3 (10.3) **[2]**	4 (6.6) **[2]**	1
	Blood creatinine increased, *n* (%)	0	0	2 (6.9) **[1]**	2 (3.3) **[1]**	0
	Glomerulonephritis membranoproliferative, *n* (%)	0	0	1 (3.4) **[1]**	1 (1.6) **[1]**	0
	Nephrotic syndrome, *n* (%)	0	1 (3.8) **[1]**	0	1 (1.6) **[1]**	1 **[1]**
	Proteinuria, *n* (%)	0	5 (19.2) **[4]**	5 (17.2) **[5]**	10 (16.4) **[9]**	1 **[1]**
	Renal failure, *n* (%)	0	0	1 (3.4) **[1]**	1 (1.6) **[1]**	0
Hepatic events	ALT increased, *n* (%)	1 (16.7)	1 (3.8) **[1]**	12 (41.4) **[11]**	14 (23.0) **[12]**	4 **[4]**
	AST increased, *n* (%)	2 (33.3)	2 (7.7) **[1]**	10 (34.5) **[9]**	14 (23.0) **[10]**	5 **[4]**
	Transaminases increased, *n* (%)	0	2 (7.7) **[2]**	2 (6.9) **[2]**	4 (6.6) **[4]**	1 **[1]**
Coagulation disorders	aPTT, *n* (%)	0	2 (7.7) **[2]**	5 (17.2) **[4]**	7 (11.5) **[6]**	1 **[1]**
	Fibrin D-dimer increased, *n* (%)	0	5 (19.2) **[4]**	9 (31.0) **[8]**	14 (23.0) **[12]**	5 **[4]**
	International normalized ratio increased, *n* (%)	1 (16.7)	1 (3.8) **[1]**	3 (10.3) **[2]**	5 (8.2) **[3]**	0
Hematological events	Anemia, *n* (%)	1 (16.7)	6 (23.1) **[2]**	9 (31.0) **[2]**	16 (26.2) **[4]**	5 **[1]**
	Leukopenia/WBC count decreased, *n* (%)	0	3 (11.5) **[3]**	0	3 (4.9) **[3]**	0
	Lymphopenia/lymphocyte count decreased, *n* (%)	0	4 (15.4) **[4]**	2 (6.9) **[2]**	6 (9.8) **[6]**	8 **[7]**
	Neutropenia/neutrophil count decreased, *n* (%)	0	2 (7.7) **[2]**	5 (17.2) **[5]**	7 (11.5) **[7]**	7 **[7]**
	Thrombocytopenia/ platelet count decreased, *n* (%)	1 (16.7) **[1]**	5 (19.2) **[5]**	13 (44.8) **[13]**	19 (31.1) **[19]**	4 **[4]**
	Any TEAE relating to WBC count, *n* (%)	0	6 (23.1) **[6]**	6 (20.7) **[6]**	12 (19.7) **[12]**	0

*ALT* alanine aminotransferase; *aPTT* activated partial thromboplastin time; *AST* aspartate aminotransferase; *N* (*n*) number of patients, *SAE* serious adverse event, *TEAE* treatment-emergent adverse event; *vp* viral particle(s); *WBC* white blood cell

^a^TEAEs are defined as any adverse event that occurs after the first administration of study treatment and through the end of the reporting period, any event that is considered treatment-related regardless of the start date of the event, or any event that is present at baseline and continues after the first dose of study treatment but worsens in intensity

^b^Selected preferred terms categorized as acute respiratory, renal, related to laboratory tests of hepatic transaminases, coagulation and hematological parameters, which were commonly related to study treatment

Numbers in bold and square brackets **[]** are the number of patients or number of events that were possibly, probably, or definitely related to study treatment

Gastrointestinal symptoms such as nausea, vomiting, and diarrhea may also be attributable to an inflammatory response to vp infusion. Nausea and vomiting were both reported by 24 patients (39.3%), with an increase in frequency seen at doses of 1 × 10^12^ vp and above. Most of these events were not considered to be treatment-related and all were CTCAE grade 1 or 2. The incidence of diarrhea was predominantly seen at doses above 3 × 10^12^ vp. Nausea and vomiting appeared dose-related, with an increase in frequency at doses of 1 × 10^12^ vp and above.

Reporting of acute respiratory symptoms encompassed acute lung injury, dyspnea, hypoxia, and interstitial lung disease. The vast majority of acute lung injury, hypoxia, and interstitial lung disease events occurred at doses above 3 × 10^12^ vp (Table 1). All but one of the six patients experiencing hypoxia had severe CTCAE grade 3 events. Dyspnea was reported across the dosing range; however, these events were mild to moderate in nature at doses of 3 × 10^12^ vp or below.

Assessment of renal function revealed that TEAEs of renal failure and acute renal injury considered treatment-related occurred at doses above 3 × 10^12^ vp. One patient developed treatment-related nephrotic syndrome after treatment with 3 × 10^12^ vp. In addition to five patients (17.2%) at doses higher than 3 × 10^12^ vp, four patients (15.4%) receiving 3 × 10^12^ vp had proteinuria considered treatment-related.

Investigations relating to changes in hepatic function (increased transaminases) were seen more commonly at doses above 3 × 10^12^ vp. These were considered treatment-related in three patients (11.5%) receiving 3 × 10^12^ vp and 12 patients (41.4%) at doses above 3 × 10^12^ vp.

The most common laboratory investigations relating to coagulation events were increased fibrin D-dimer levels and prolonged activated partial thromboplastin time. These events were not reported at doses below 3 × 10^12^ vp but were reported in 14 (23%) and seven patients (11.5%) at doses of 3 × 10^12^ and above, which were considered treatment-related in 12 (19.7%) and six patients (9.8%), respectively. Anemia was observed across the dose range but most cases were not considered treatment-related. Falls in total white blood cell count were seen at doses of 1 × 10^12^ vp and above, and the proportion of patients with neutropenia increased with dose (two patients [7.7%] at doses of ≤ 3 × 10^12^ vp compared with five patients [17.2%] at doses > 3 × 10^12^ vp), and all were considered treatment-related. Likewise, decreased platelet counts increased with dose (six patients [18.8%] at doses of ≤ 3 × 10^12^ vp compared with 13 patients [44.8%]) at doses > 3 × 10^12^ vp). Falls in platelet count (reported as either thrombocytopenia or platelet count decreased) were observed in 19 patients (31.1%) and considered treatment-related in all patients (Table 1).

Intestinal obstruction (reported as gastrointestinal obstruction, small intestinal obstruction, and intestinal obstruction) was reported in seven patients, all at doses of 3 × 10^12^ vp and above. In all cases, there were predisposing factors but it was considered treatment-related in two patients treated as part of phase 1b. Abdominal pain and abdominal pain upper were reported by 13 patients (21.3%), predominantly at doses of 3 × 10^12^ vp and above. These events were considered treatment-related in five patients.

### Enadenotucirev pharmacokinetics

**Fig. 2:**
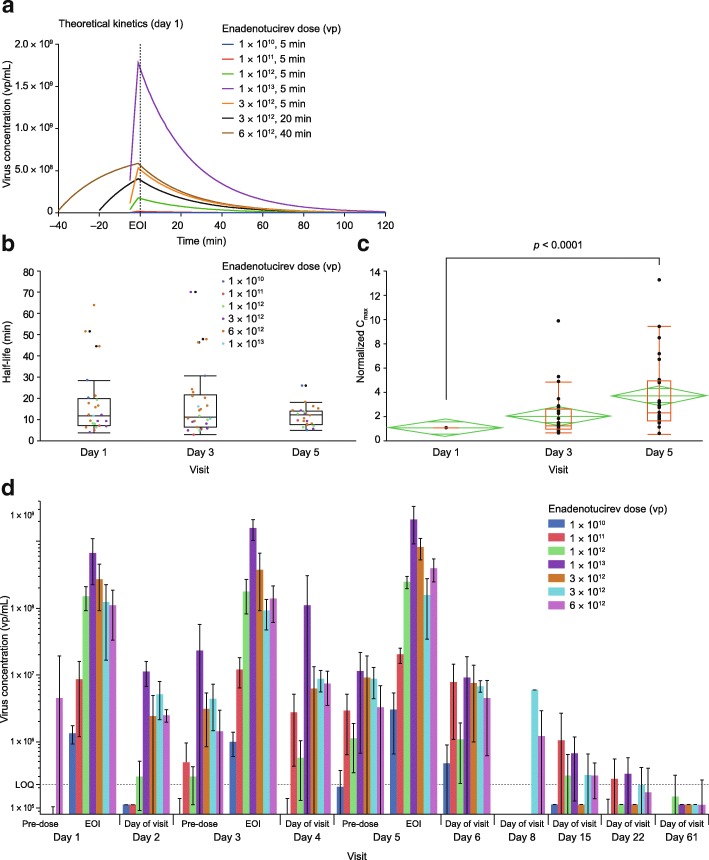
Blood viral kinetics and mean enadenotucirev concentration in blood by cohort. **a** Theoretical day 1 kinetics of enadenotucirev. **b** Box plot showing the mean (and 95% CI) half-life across dosing cohorts of EVOLVE phase 1a on dosing days 1, 3, and 5. **c** Fold change increase in C_max_ across dose administrations. The red box represents outlier box plot quantile analysis, and the green diamond represents mean and 95% CI across dosing cohorts of EVOLVE phase 1a on dosing days 1, 3, and 5. **d** Mean (± SD) enadenotucirev blood concentration by cohort. *CI* confidence interval, *C*_*max*_ maximum serum concentration, *EOI* end of infusion, *EVOLVE* EValuating OncoLytic Virus Efficacy, *LOQ* level of quantification, *SD* standard deviation, *vp* viral particle(s)

In line with data from theoretical modeling (Fig. 2a), upon enadenotucirev infusion, blood virus concentration kinetics fit to a one-phase decay with a dose-independent alpha half-life of 16.7 min. There was no significant change in mean half-life of virus clearance observed on day 1 in response to increasing doses (Additional file 2: Figure S1a). Thus, it was concluded that half-life is dose-independent over the study range. Subsequent comparison of doses showed no significant differences in alpha half-life on days 1, 3, and 5 (Fig. 2b), when repeated doses were administered on either Q3W or Q1W infusion schedules (Additional file 2: Figure S1b), or at different doses (Additional file 2: Figure S1c). There was, however, a significant increase in maximum serum concentration (C_max_) between days 1 and 5 (Fig. 2c; *p* < 0.0001) of cycle 1. By day 61, genomic DNA levels had dropped to below the level of quantification (LOQ) in 26/28 (92.9%) blood samples (Fig. 2d).

The qPCR signal may, however, comprise infectious, non-infectious, and/or broken-down viral products containing the relevant DNA sequence. Evidence of active virus came from an additional investigation of one patient with mCRC, who had abdominal wall metastases biopsied 39 days after the last enadenotucirev dose (following four cycles of 6 × 10^12^ vp in a Q3W schedule), which showed extensive areas of cell necrosis and evidence of viral replication (Additional file 2: Figure S2). These findings are consistent with results from the MOA study [15].

### Post-infusion viral shedding

**Fig. 3:**
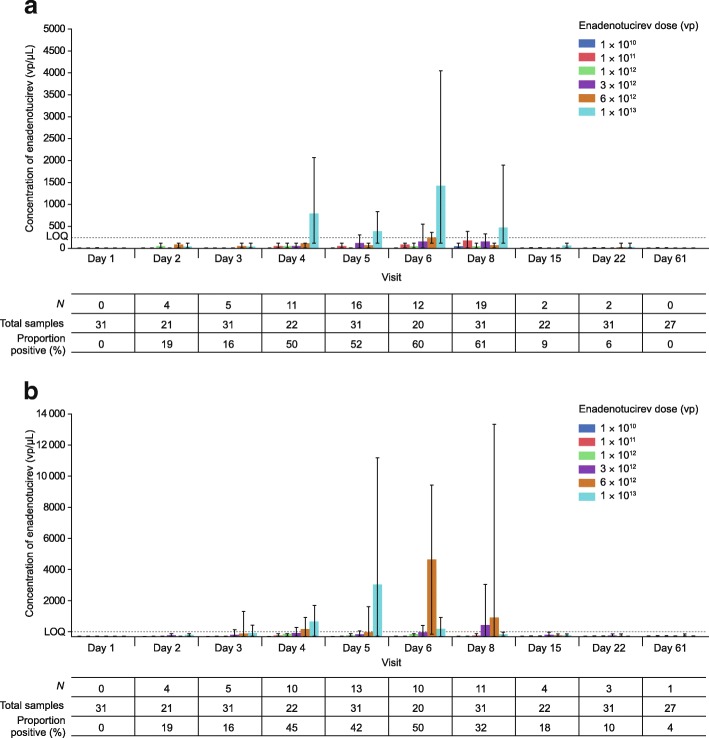
Post-infusion viral shedding during phase 1a. **a** Mean buccal shedding as quantified by qPCR with solid bars representing the mean and error bars representing the range observed. The number of patients in whom buccal shedding was detected (*N*; > 0 vp/μL) is represented by *N*. The total number of samples represents the number of samples taken at each time point. The proportion positive is the percentage of samples in which buccal shedding was detected across all cohorts. **b** Mean rectal shedding as quantified by qPCR with bars representing the mean and error bars the range observed. The number of patients in whom rectal shedding was detected (*N*; > 0 vp/μL) is represented by *N*. The total number of samples represents the number of samples taken at each time point. The proportion positive is the percentage of samples in which buccal shedding was detected across all cohorts. *LOQ* level of quantification, *N* number, *qPCR* quantitative polymerase chain reaction, *vp* viral particle(s)

Buccal shedding (phase 1a) was detected at all doses in all but six patients, and shedding frequency and concentration were generally related to infusion concentration (Fig. 3a). Shedding was most common between day 6 and day 8, which is equivalent to 24–72 h after the final dose of enadenotucirev.

Rectal shedding (phase 1a) was related to dose level, detected in all but the lowest dosing cohort (1 × 10^10^ vp), and overall in 17 of the 31 patients (Fig. 3b). Viral DNA was most commonly observed in the rectal swabs between day 4 and day 8 (24–72 h following the final dose of enadenotucirev).

Urine shedding (phase 1a and phase 1b) could be detected in all samples; however, for phase 1a, this was only at low concentrations because no legitimate samples gave a concentration above the LOQ (Additional file 2: Figure S3a). In phase 1b, shedding was observed only above the LOQ in two samples (Additional file 2: Figure S3b). This was seen in one patient who had UCC and had not had their bladder removed. A relatively large concentration of viral DNA was seen in the urine 48 h after initial dosing, which may have been the result of viral replication in a penetrating tumor.

### Antibody response to enadenotucirev in phase 1b

**Fig. 4:**
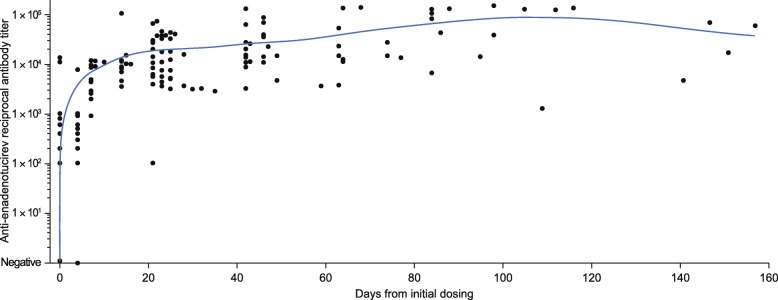
Summary of anti-enadenotucirev antibody response. Scatter plot of anti-enadenotucirev antibody titer over time, with the blue line representing the moving mean

Before dosing, most patients had no or very low levels of anti-enadenotucirev antibodies. Following enadenotucirev infusion, all patients showed an increase in antibody titer from baseline, irrespective of dose and schedule (Fig. 4). This increase plateaued by day 20 (for all patient groups, individual variations were noted) and was sustained thereafter.

To evaluate the potential impact of this antibody response on enadenotucirev infectivity in the blood, post-infusion samples were tested in a virus infectivity assay (Table 2 and Additional file 2: Figure S4). Strong virus infectivity was observed in all (14/14) samples collected following infusion on day 1, cycle 1. Viable virus infectivity was also observed in samples from 12/15 patients (80%) on day 1, cycle 2 (day 22). Subsequent assessment on day 1, cycle 3 revealed that samples from fewer patients (3/12 [25%]) displayed infectivity (day 43).

**Table 2:** Summary table of results from viral infectivity assay

Time point	Negative,^a^ *n* (%)	Positive,^a^ *n* (%)			Total number of patient samples analyzed
		Cells stain positive for virus	Plaques in monolayer	Complete/ partial monolayer destruction or quantifiable	
Cycle 1, day 1 (day 0)	0 (0)	0	0	14 (100)	14
Cycle 2, day 1 (day 22)	3 (20)	0	4 (26.7)	8 (53.3)	15
Cycle 3, day 1 (day 43)	9 (75)	3 (25)	0	0	12

^a^Example images for each level of infectivity are provided in Additional file 2: Figure S5

### Cytokine response to enadenotucirev

**Fig. 5:**
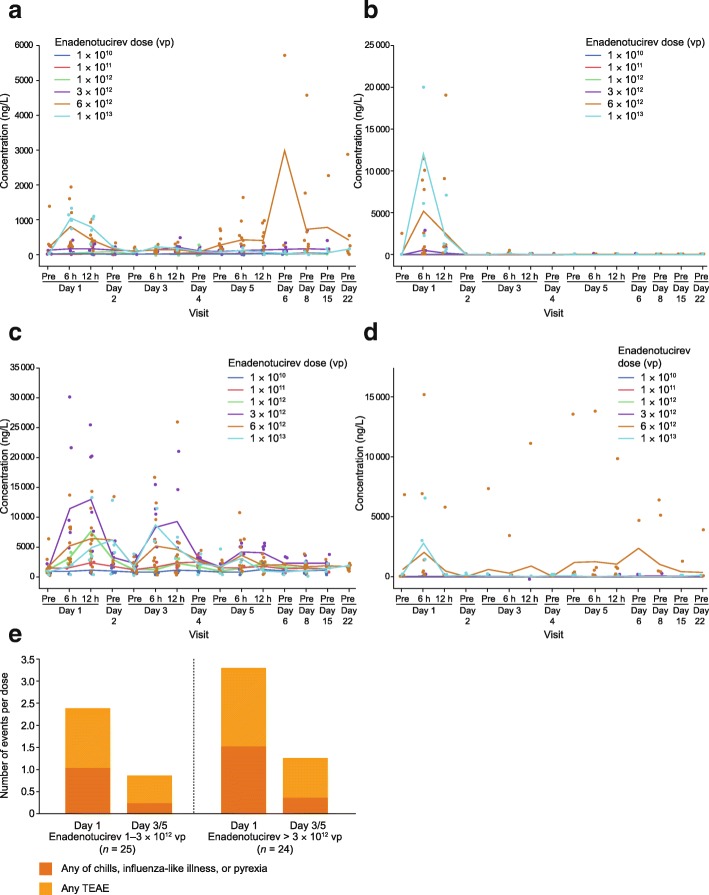
Cytokine levels in the blood by infusion dose during phase 1a. **a** Mean concentration of IFN-γ by dose. **b** Mean concentration of IL-6 by dose. **c** Mean concentration of MCP-1 by dose. **d** Mean concentration of TNF-α by dose. **e** Frequency of TEAEs (including any of chills, influenza-like illness, and pyrexia) per dose occurring within 24 h of infusion on cycle 1, day 1 or following infusion on day 3/5. *IFN* interferon, *IL* interleukin, *MCP* monocyte chemoattractant protein, *TEAE* treatment-emergent adverse event, *TNF* tumor necrosis factor, *vp* viral particle(s)

Analysis was limited to the cytokines that produced the most robust response on cycle 1, day 1 (i.e. interferon [IFN]-γ, interleukin [IL]-6, monocyte chemoattractant protein [MCP]-1, and tumor necrosis factor [TNF]-α). Transient increases were observed after the first dose in most patients, and these were dose-dependent (Fig. 5a–d). When elevated cytokine levels were observed in a patient following the initial dose, subsequent administrations on days 3 and 5 generally resulted in lower responses (Fig. 5a–d). These lower cytokine responses extended to multiple repeat-dose administrations performed within a 2–3-day window of previous doses, and continued, albeit to a lesser extent, if repeat dosing was performed within 7–17 days. This suppressive effect was diminished beyond cycle 2, with mean cytokine responses increasing more following 17-day (Q3W schedule) than 7-day (Q1W schedule) delays in treatment (Additional file 2: Figure S5a, c). This was, however, not observed across all cytokines analyzed (Additional file 2: Figure S5b, d).

Enadenotucirev dosing on subsequent days (i.e. day 3/5) was associated with a reduced frequency of TEAEs per dose during cycle 1 (Fig. 5e). A reduction was also observed in the total number of events on cycle 2, day 1, compared with cycle 1, day 1 (Additional file 2: Figure S5e).

### Enadenotucirev efficacy

This phase 1 study was not designed to assess efficacy, but stable disease lasting for more than 12 weeks was recorded in five patients by both independent and investigator assessment (per RECIST v1; Additional file 1: Table S2).

## Discussion

EVOLVE was the first study initiated, although the second to report, within the ongoing enadenotucirev clinical program, initially planned as a phase 1/2 multicenter, open-label clinical study investigating single- and repeat-cycle dosing of enadenotucirev monotherapy. The disposition of patients observed was not entirely unexpected in this heavily pre-treated, typical phase 1 patient population. By the end of the study, the MTD was determined to be 3 × 10^12^ vp, irrespective of infusion duration or dosing schedule. Most patients at this MTD experienced inflammatory influenza-like events, which are typical of oncolytic virus exposure, within 24 h of dosing; these, however, were manageable with appropriate treatment.

The data from EVOLVE provide additional insights into enadenotucirev dosing that relate to the safety and tolerability of repeat-dose scheduling. Of note was the reduced frequency of TEAEs during subsequent treatment cycles compared with those observed within 24 h of cycle 1, day 1. The potential to schedule a lower dose on day 1 followed by a higher dose on days 3 and 5 is therefore worthy of further investigation. During previous studies of systemically administered viruses, changes in the cytokine profile (notably IL-6) have correlated with signs of toxicity [20]. Herein, increases in circulating cytokine levels (notably IFN-γ, IL-6, MCP-1, and TNF-α) were observed following the first infusion, but were attenuated in response to further infusions (days 3 and 5). These attenuated cytokine responses also confirm preclinical observations that informed the dosing schedule used as part of this study [17]. These findings were also consistent across Q3W and Q1W dosing schedules during the first three treatment cycles. Therefore, management of early AE manifestations by setting the initial dose to be lower than subsequent doses may prove advantageous.

The EVOLVE study represents the first assessment of the pharmacokinetic behavior of a systemically administered group B adenovirus in humans. The use of a clustered administration regimen (three doses given in 5 days) resulted in a modest increase in C_max_ on day 5 relative to after the first dose. The increase was greater than can be explained by accumulation alone and is consistent with a response to the initial virus dose leading to suppression of virus clearance activity by cells of the macrophage lineage, such as Kupffer cells in the liver. However, no change in half-life was observed, which may be due to the sensitivity of the assay and sampling schedule used. This increase in circulating virus loading occurred in addition to the diminished induction of blood cytokine responses and side effect manifestation. These findings are consistent with the hypothesis that innate immune cells can be desensitized, allowing improved delivery and fewer side effects with subsequent doses.

The EVOLVE study provided further evidence of the advantages of enadenotucirev in the context of IV dosing with oncolytic viruses. Consistent with previous reports of group B adenoviruses in the general population [18, 19], a low prevalence of neutralizing antibodies was observed before initial dosing with enadenotucirev. An antibody response was observed in most patients during the EVOLVE study, with antibody titers plateauing approximately 20 days after first exposure. Despite this antibody response, live virus could still be detected in the blood of most participants following the second cycle, indicating virus availability. However, the virus activity was lower at cycle 2 than cycle 1 and declined further by cycle 3, suggesting that the overall affinity of the antibody response might be increasing, leading to an increase in overall neutralizing activity. However, it is not known whether, or to what extent these antibodies may interfere with virus delivery to and activity within tumors. Furthermore, detection of enadenotucirev in the metastatic lesion presented herein and findings from our previous MOA study suggest persistence within the tumor [15], thereby potentially reducing the need for frequent repeat dosing.

Taken together, these observations will inform the ongoing clinical program evaluating enadenotucirev (1–3 × 10^12^ vp) in combination with additional cancer therapeutics. Further investigations into the potential of enadenotucirev will take advantage of this unique platform to incorporate transgene add-ons and use systemic dosing to deliver therapeutic agents to the tumor microenvironment. Further modifications have utilized matrix-degrading enzymes to remove interstitial barriers and to improve the spread of enadenotucirev between tumor ‘islands’ [21]. Additionally, enadenotucirev has been engineered to express a range of biotherapeutic molecules singly and in combination [14]. One example is the expression of a bispecific T-cell engager (BiTE), facilitating the clustering and activation of CD4 and CD8+ T cells [22]. A major advantage of this system is that BiTE (or other transgene) transcription can be supressed until activation of the virus’ major late promoter, conferring specificity to cancer cells in which viral replication is occurring.

One factor that is common to the most successful oncolytic viruses developed to date is the capacity to provoke anticancer immune responses. Mechanisms by which tissues are protected from immune response hyperactivation can, however, be co-opted by tumor cells to avoid destruction [23, 24]. Indeed, signaling adaptations that occur upon recognition of its ligand by programmed cell death protein 1 (PD-1) within the tumor microenvironment represent a major mechanism of immune resistance [25, 26]. The evidence generated during both the MOA and EVOLVE studies has highlighted the recruitment of CD8+ T cells to the tumor microenvironment, strongly suggesting the potential benefit of combining enadenotucirev with PD-1 checkpoint blockade [15]. Indeed, such synergy has recently been shown in a study using a combination of talimogene laherparepvec and the PD-1 inhibitor pembrolizumab in patients with advanced melanoma [24]. The safety and tolerability of IV enadenotucirev combined with the PD-1 inhibitor nivolumab is currently being tested for the treatment of epithelial carcinomas as part of the phase 1 trial SPICE (NCT02636036) [27].

With the knowledge that most cancer patients receive standard-of-care chemotherapies, exploring the potential synergy between such agents and enadenotucirev may prove clinically relevant. The potential of viral–chemotherapy combinations has also been demonstrated, with paclitaxel in particular shown to increase the replicative potential of adenoviruses [28]. Therefore, the combination of enadenotucirev and paclitaxel is currently being tested for the treatment of platinum-resistant ovarian cancer as part of the phase 1 OCTAVE study (NCT02028117) [29].

## Conclusions

Taken together, the results from EVOLVE showed that enadenotucirev monotherapy can be administered in a single cycle or repeated cycles with manageable tolerability. These findings also confirm previous observations of enadenotucirev’s low immunogenicity, short half-life in the circulation, and consistent viral clearance over repeated cycles. While only limited information was gained with respect to the antitumor activity of enadenotucirev monotherapy, the safety information will inform future studies utilizing systemic delivery of combinatorial therapies.

## Additional files

Additional file 1: **Table S1.** Summary table of dosing cohorts. **Table S2.** Independent assessment of best overall response (per RECIST). (DOCX 43 kb)

Additional file 2: **Figure S1.** Comparison of viral kinetics following different doses and schedules. **a** Scatter plot of calculated half-life of enadenotucirev in blood in cycle 1, day 1 (phase 1a) by dose. The red box represents outlier box plot quantile analysis, and the green diamonds represent mean and 95% CI at each dose. **b** Scatter plot of viral clearance half-life by schedule in phase 1b. The horizontal line represents the mean of each schedule. **c** Scatter plot of viral clearance half-life for each patient on each visit, coloured by dose (horizontal lines represent the mean half-life for each dose at each time point). **Figure S2.** Biopsy of skin metastasis after treatment with enadenotucirev. Skin biopsy taken after four cycles of enadenotucirev dosing (6 × 10^12^ vp, Q3W), 107 days after first exposure (39 days after final dose). **Figure S3.** Mean urine viral shedding. As quantified by qPCR with bars representing the mean and error bars representing **a** the range observed by dose during phase 1a and **b** by schedule in phase 1b. **Figure S4.** Representative viral infectivity assay images. Images taken during the viral infectivity assay displaying **a** negative, **b** cells stained positive for virus, **c** plaques in monolayer, and **d** complete/partial monolayer destruction or quantifiable scoring. **Figure S5.** Cytokine levels in the blood by dosing schedule during phase 1b. As measured using a Luminex bead-based multiplex assay. **a** Mean concentration of IFN-γ by schedule. **b** Mean concentration of IL-6 by schedule. **c** Mean MCP-1 concentration by schedule. **d** Mean TNF-α concentration by schedule. **e** Total number of TEAEs of interest (any of chills, influenza-like illness, and pyrexia) occurring within 24 h of infusion across cycles. (DOCX 3593 kb)

## Abbreviations

AE: Adverse event
BiTE: Bispecific T-cell engager
CEC: Clinical Events Committee
C_max_: Maximum serum concentration
CRC: Colorectal cancer
CTCAE: Common Terminology Criteria for Adverse Events
DLT: Dose-limiting toxicity
DSMC: Data and Safety Monitoring Committee
EOI: End of infusion
EVOLVE: EValuating OncoLytic Virus Efficacy
IFN: Interferon
IL: Interleukin
IT: Intratumoral
IV: Intravenous
LOQ: Level of quantification
MCP: Monocyte chemoattractant protein
mCRC: Metastatic colorectal cancer
MOA: Mechanism of Action
MTD: Maximum tolerated dose
PD-1: Programmed cell death protein 1
Q1W: Weekly
Q3W: 3-weekly
qPCR: Quantitative polymerase chain reaction
RECIST: Response Evaluation Criteria in Solid Tumors
TEAE: Treatment-emergent adverse event
TNF: Tumor necrosis factor
UCC: Urothelial cell carcinoma
vp: Viral particle(s)
