# Supplementary material for: A phase 1 dose escalation study of the oncolytic adenovirus enadenotucirev, administered intravenously to patients with epithelial solid tumors (EVOLVE)
Source: J Immunother Cancer. 2019 Jan 28;7:20. doi: 10.1186/s40425-019-0510-7 (PMC6348630; doi:10.1186/s40425-019-0510-7)
Supplement: Supplementary file 1 — Table S1. Summary table of dosing cohorts. Table S2. Independent assessment of best overall response (per RECIST). (DOCX 43 kb) [file 40425_2019_510_MOESM1_ESM.docx]

# Supplementary materials

Table S1 Summary table of dosing cohorts

|  | Dose^a^ (vp)/ infusion duration | Schedule | Number of patients | Tumor type (*n*) |
| --- | --- | --- | --- | --- |
| Phase 1a  (*N* = 37) | 1 × 10^10^/5 min | Single cycle | 3 | Oropharynx, liver, stomach |
|  | 1 × 10^11^/5 min | Single cycle | 3 | Gall bladder, parotid, CRC |
|  | 1 × 10^12^/5 min | Single cycle | 3 | Breast, CRC (2) |
|  | 1 × 10^13^/5 min  (MAD) | Single cycle | 4 | Salivary gland, parotid, CRC (2) |
|  | 3 × 10^12^/5 min | Single cycle | 3 | CRC (3) |
|  | 3 × 10^12^/20 min | Single cycle | 3 | CRC (3) |
|  | 6 × 10^12^/40 min | Single cycle | 3 | CRC (3) |
|  | 6 × 10^12^/40 min | Single cycle | 9 | CRC (9) – dose expansion cohort |
|  | 6 × 10^12^/40 min | Q3W^b^ | 6 | CRC (6) – repeat cycle cohort |
| Phase 1b  (*N* = 24) | 6 × 10^12^/40 min | Q1W^c^ | 3 | CRC (2), UCC |
|  | 3 × 10^12^/15 min^c,d^ | Q1W | 8 | CRC (4), UCC (4) |
|  | 6 × 10^12^/40 min | Q3W | 4 | CRC (3), UCC |
|  | 3 × 10^12^/15 min^d^ | Q3W | 6 | CRC (3), UCC (3) |
|  | 1 × 10^12^/5 min | Q3W | 3 | CRC (3) |

CRC, colorectal cancer; MAD, maximum administered dose; Q1W, weekly; Q3W, 3-weekly; UCC, urothelial cell carcinoma; vp, viral particle(s)

^a^Dose administered on each of days 1, 3, and 5 of each cycle (single cycle and Q3W)

^b^Patients received three cycles of study treatment, with the possibility of a fourth cycle if the first three cycles were well tolerated. Five of six patients received three cycles, one of six received four cycles

^c^Dose administered on day 1, 3, 5, and then day 8, and weekly thereafter (Q1W)

^d^Infusion duration reduced to 15 min (after January 22, 2015) to achieve an infusion rate of 2 × 10^11^ vp/min in line with previous cohorts

Table S2 Independent assessment of best overall response (per RECIST)

|  | | Number of patients, *n* (%) | | | | | | | |
| --- | --- | --- | --- | --- | --- | --- | --- | --- | --- |
|  |  | Investigator | | | | Independent | | | |
|  |  | PD | SD | PR | NE | PD | SD | PR | NE |
| Phase 1a (*N* = 37) | | 21 (56.8) | 14 (37.8) **[4]** | 0 | 2 (5.4) | 19 (51.4) | 15 (40.5) **[4]** | 0 | 3 (8.1)^a^ |
| Phase 1b (*N* = 24) | Dose assigned (vp) |  | | | | | | | |
| 3 | 1 × 10^12^ | 3 (100) | 0 | 0 | 0 | 2 (66.7) | 0 | 0 | 1 (33.3) |
| 14 | 3 × 10^12^ | 8 (57.1) | 2 (14.3) | 1 (7.1) **[1]** | 3 (21.4) | 9 (64.3) | 2 (14.3) **[1]** | 0 | 3 (21.4) |
| 7 | 6 × 10^12^ | 6 (85.7) | 0 | 0 | 1 (14.3) | 4 (57.1) | 1 (14.3) | 0 | 2 (28.6) |

NE, not evaluable; PD, progressive disease; PR, partial response; RECIST, Response Evaluation Criteria in Solid Tumors; SD, stable disease; vp, viral particle(s)

^a^No assessment

Numbers in bold and square brackets **[ ]** are those with stable disease for ≥ 12 weeks
