# Supplementary material for: A phase 1 dose escalation study of the oncolytic adenovirus enadenotucirev, administered intravenously to patients with epithelial solid tumors (EVOLVE)
Source: J Immunother Cancer. 2019 Jan 28;7:20. doi: 10.1186/s40425-019-0510-7 (PMC6348630; doi:10.1186/s40425-019-0510-7)
Supplement: Supplementary file 2 — Figure S1. Comparison of viral kinetics following different doses and schedules. a Scatter plot of calculated half-life of enadenotucirev in blood in cycle 1, day 1 (phase 1a) by dose. The red box represents outlier box plot quantile analysis, and the green diamonds represent mean and 95% CI at each dose. b Scatter plot of viral clearance half-life by schedule in phase 1b. The horizontal line represents the mean of each schedule. c Scatter plot of viral clearance half-life for each patient on each visit, coloured by dose (horizontal lines represent the mean half-life for each dose at each time point). Figure S2. Biopsy of skin metastasis after treatment with enadenotucirev. Skin biopsy taken after four cycles of enadenotucirev dosing (6 × 10^12^ vp, Q3W), 107 days after first exposure (39 days after final dose). Figure S3. Mean urine viral shedding. As quantified by qPCR with bars representing the mean and error bars representing a the range observed by dose during phase 1a and b by schedule in phase 1b. Figure S4. Representative viral infectivity assay images. Images taken during the viral infectivity assay displaying a negative, b cells stained positive for virus, c plaques in monolayer, and d complete/partial monolayer destruction or quantifiable scoring. Figure S5. Cytokine levels in the blood by dosing schedule during phase 1b. As measured using a Luminex bead-based multiplex assay. a Mean concentration of IFN-γ by schedule. b Mean concentration of IL-6 by schedule. c Mean MCP-1 concentration by schedule. d Mean TNF-α concentration by schedule. e Total number of TEAEs of interest (any of chills, influenza-like illness, and pyrexia) occurring within 24 h of infusion across cycles. (DOCX 3593 kb) [file 40425_2019_510_MOESM2_ESM.docx]

# Supplementary materials

Figure S1 Comparison of viral kinetics following different doses and schedules


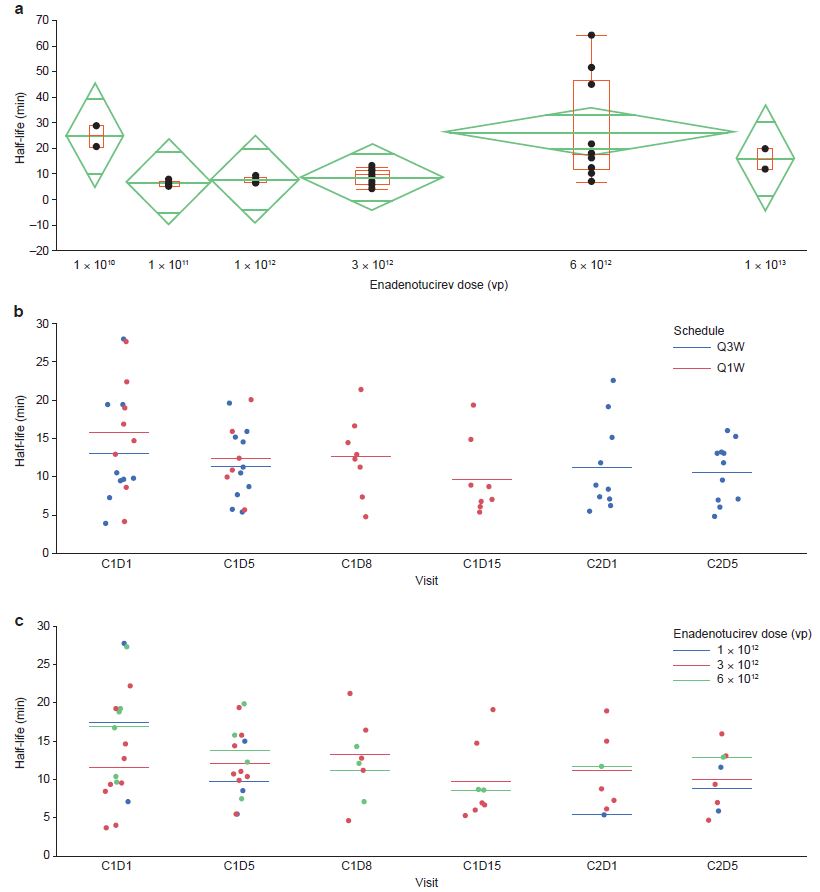


Comparing viral kinetics on different doses and schedules. **a** Scatter plot of calculated half-life of enadenotucirev in blood in cycle 1, day 1 (phase 1a) by dose. The red box represents outlier box plot quantile analysis, and the green diamonds represent mean and 95% CI at each dose. **b** Scatter plot of viral clearance half-life by schedule in phase 1b. The horizontal line represents the mean of each schedule. **c** Scatter plot of viral clearance half-life for each patient on each visit, coloured by dose (horizontal lines represent the mean half-life for each dose at each time point)

C, cycle; CI, confidence interval; D, day; Q1W, weekly schedule; Q3W, 3-weekly schedule; vp, viral particle(s)

Figure S2 Biopsy of skin metastasis after treatment with enadenotucirev

**
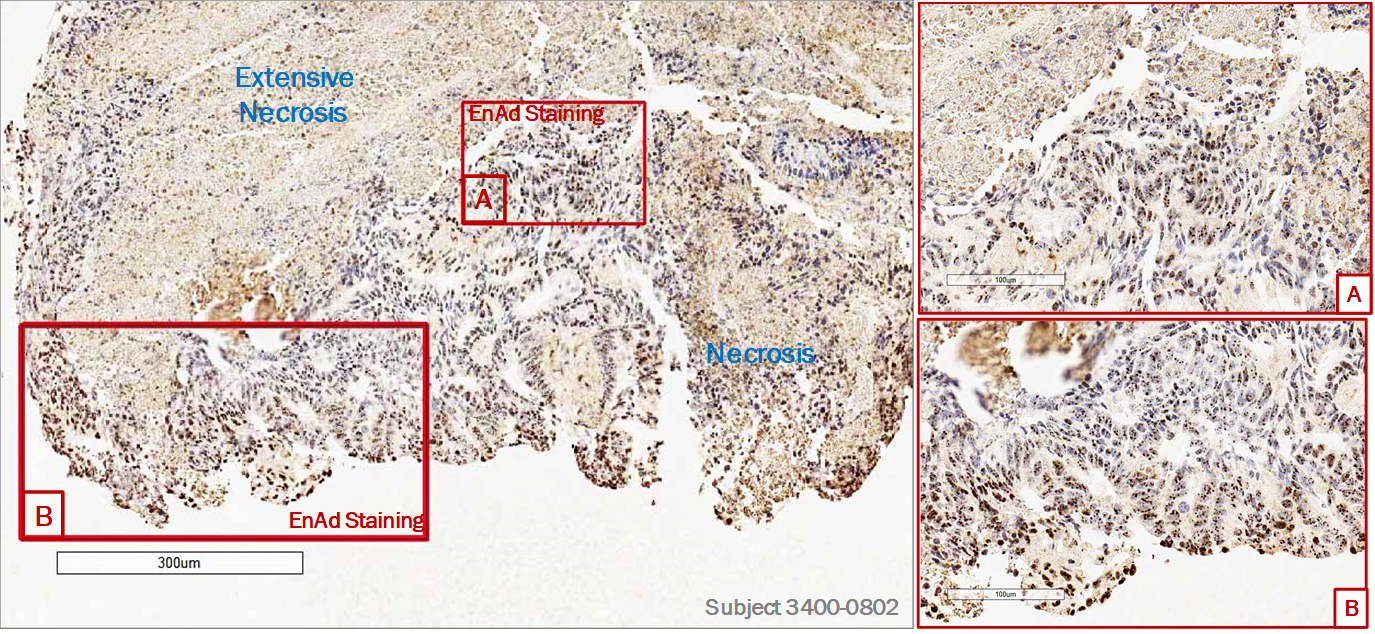
**

Biopsy of skin metastasis after treatment with enadenotucirev. Skin biopsy taken after four cycles of enadenotucirev dosing (6 × 10^12^ vp, Q3W), 107 days after first exposure (39 days after final dose)

Q3W, 3-weekly schedule; vp, viral particle(s)

Figure S3 Mean urine viral shedding


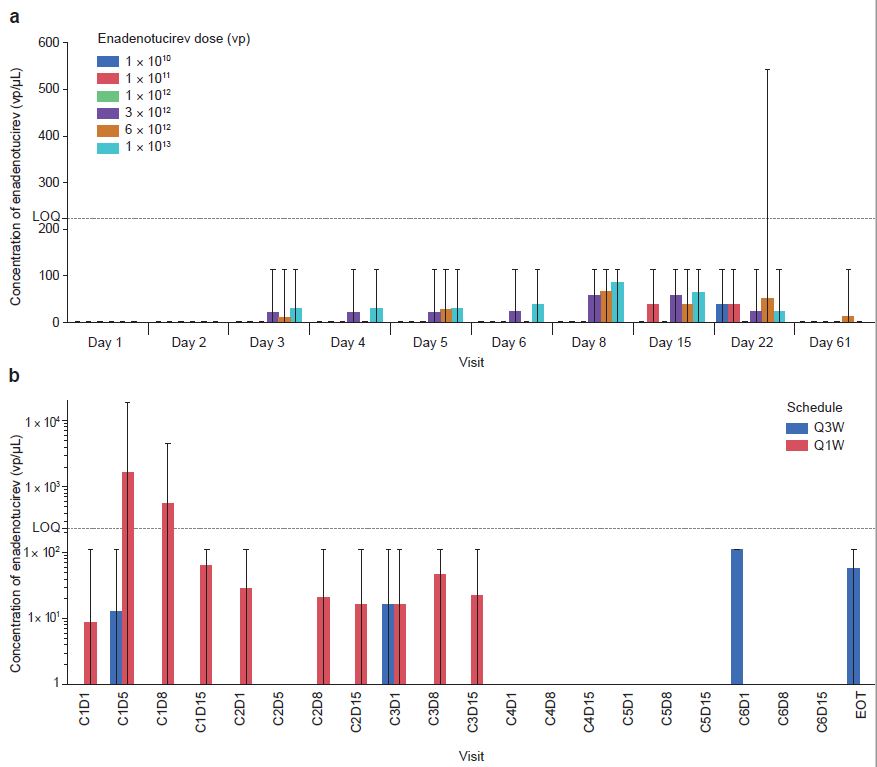


Mean urine viral shedding. As quantified by qPCR with bars representing the mean and error bars representing **a** the range observed by dose during phase 1a and **b** by schedule in phase 1b

C, cycle; D, day; EOT, end of treatment; LOQ, level of quantification; Q1W, weekly schedule; Q3W, 3-weekly schedule; qPCR, quantitative polymerase chain reaction; vp, viral particle(s)

Figure S4 Representative viral infectivity assay images


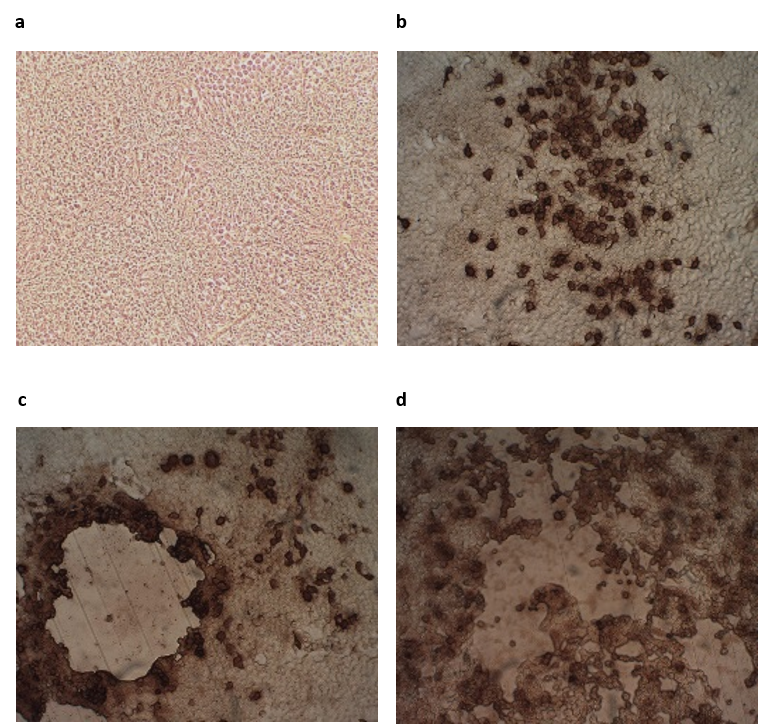


Representative viral infectivity assay images. Images taken during the viral infectivity assay displaying **a** negative, **b** cells stained positive for virus, **c** plaques in monolayer, and **d** complete/partial monolayer destruction or quantifiable scoring

Figure S5 Cytokine levels in the blood by dosing schedule during phase 1b


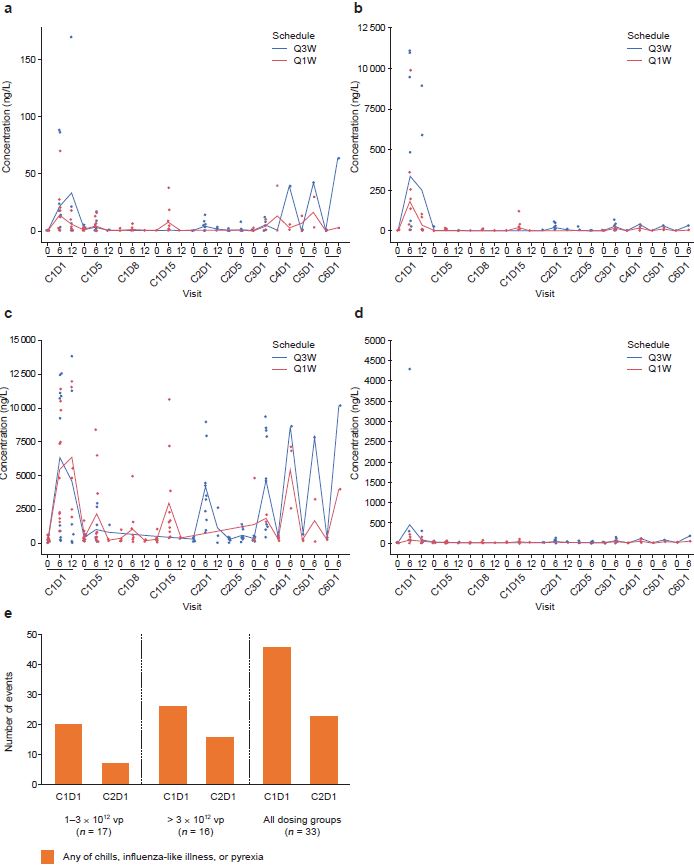


Cytokine levels in the blood by dosing schedule during phase 1b. As measured using a Luminex bead-based multiplex assay. **a** Mean concentration of IFN-γ by schedule. **b** Mean concentration of IL-6 by schedule. **c** Mean MCP-1 concentration by schedule. **d** Mean TNF-α concentration by schedule. **e** Total number of TEAEs of interest (any of chills, influenza-like illness, and pyrexia) occurring within 24 hours of infusion across cycles

C, cycle; D, day; IFN, interferon; IL, interleukin; MCP, monocyte chemoattractant protein; Q1W, weekly schedule; Q3W, 3‑weekly schedule; TEAE, treatment-emergent adverse event; TNF, tumor necrosis factor; vp, viral particle(s)
